# Understanding of Nanophase Separation and Hydrophilic Morphology in Nafion and SPEEK Membranes: A Combined Experimental and Theoretical Studies

**DOI:** 10.3390/nano9060869

**Published:** 2019-06-07

**Authors:** Rujie Wang, Shanshan Liu, Lidong Wang, Ming Li, Chong Gao

**Affiliations:** 1 Hebei Key Lab of Power Plant Flue Gas Multi-Pollutants Control, Department of Environmental Science and Engineering, North China Electric Power University, Baoding 071003, China; rujiewang@gmail.com (R.W.); tracy36656@outlook.com (S.L.); ming2999@126.com (M.L.); 18830252613@163.com (C.G.); 2 MOE Key Laboratory of Resources and Environmental Systems Optimization, College of Environmental Science and Engineering, North China Electric Power University, Beijing 102206, China

**Keywords:** nanophase separation, molecular dynamics, proton exchange membrane, proton conductive channel, fuel cell

## Abstract

The understanding of the relationship between the chemical structure and the hydrophilic structure is crucial for the designing of high-performance PEMs. Comparative studies in typical Nafion and sulfonated poly (ether ether ketone) (SPEEK) were performed using a combined experimental and theoretical method. SPEEK showed suppressed fuel crossover and good mechanical property but low water uptake, weak phase separation, and inadequate proton conductivity. Molecular dynamics (MD) simulation approaches were employed to get a molecular-level understanding of the structure–property relationship of SPEEK and Nafion membranes. In SPEEK membranes, the local aggregation of hydrophilic clusters is worse, and much stronger electrostatic interaction between O_s_–H_h_ was verified, resulting in less delocalized free H_3_O^+^ and much lower D_H3O+_. In addition, the probability of H_2_O–H_3_O^+^ association varied with water content. Particularly, SPEEK exhibited much lower H_9_O_4_^+^ probability at various relative water contents, leading to lower structural diffusivity than Nafion. Eventually, SPEEK possessed low vehicular and structural diffusivities, which resulted in a low proton conductivity. The results indicated that the structure of hydrated hydronium complexes would deform to adapt the confining hydrophilic channels. The confinement effect on diffusion of H_2_O and H_3_O^+^ is influenced by the water content and the hydrophilic morphologies. This study provided a new insight into the exploration of high-performance membranes in fuel cell.

## 1. Introduction

Proton exchange membrane fuel cell (PEMFC) is a type of renewable and sustainable electricity generation technology with the advantage of high efficiency and zero emission [1,2,3]. As a performance–limiting component of PEMFC, proton exchange membrane (PEM) plays an important role in maintaining high intrinsic proton conductivity and insulating electrode [4,5]. Perfluorosulfonic acid (PFSA) ionomers, in particular Nafion, composed of a hydrophobic polytetrafluoroethylene (PTFE) backbone with pendant hydrophilic side chains terminated by a sulfonic acid, are the most widely used benchmark PEM materials because of good stability and high proton conductivity [6,7,8]. However, they are limited by their low operation temperature, high cost, and high fuel crossover [9,10,11]. To address the problems, sulfonated nonfluoroniated aromatic materials, such as poly(phenylene)s [12,13], polyphenylenes [14], polyimides [15], poly (arylene ether sulfone) [16,17], and poly (ether ether ketone) (SPEEK) [18] have been proposed with good chemical/thermal stability, low cost, and high mechanical stability. However, poor hydrophilic/hydrophobic phase separation and insufficient proton conductivity were reported [19,20,21]. It is well known that the macroscopic properties, especially proton conductivity, were dominated by the chemical structure and nanophase separation morphology [22,23,24]. The understanding of the relationship between the chemical structure and the hydrophilic structure is crucial for the design of novel high-performance PEM.

Polymers with different chemical structures presented different hydrophilic clusters and different proton transport phenomena [25,26]. It is well established that the polymer membranes consist of nanophase separation of a hydrophobic matrix and hydrophilic clusters, wherein proton ions transport through the hydrophilic domain composed of a water molecule, proton, and pendant sulfonate group (-SO_3_^−^) [27,28]. At the molecular scale, proton transport in confined hydrophilic channels includes structural diffusion by proton hopping or a Grotthuss mechanism, and vehicular diffusion by a diffusion mechanism [29,30]. Molecular simulations have been applied to advance the understanding of the PEM structure and the proton transport at a molecular level [31,32,33,34,35,36,37]. It is reported that water transport was dependent on the structure of polymer chain and the water content [38], and the vehicular transport of hydronium ions was influenced by the electrostatic interaction between the hydronium and the pendant -SO_3_^−^ [39,40]. Zhang et al. found that the membrane blockage significantly reduced water diffusivity in the membrane than bulk water [41]. Mabuchi et al. reported that the Grotthuss mechanism became predominant when the number of water molecules per -SO_3_^−^ (*λ*) surpassed 5.6 [42]. In spite of these valuable experimental and theoretical studies, the detailed mechanism of proton transport in a hydrated domain remains unclear. Moreover, it is crucial to understand the influence of different polymer materials on the hydrophilic cluster structure and the proton transport properties.

On the basis of the aforementioned consideration, experiment and molecular dynamics (MD) simulation were used to study the structure–property relationship using two typical PEMs, perfluorinated Nafion and nonfluoroniated SPEEK. First, their water uptake, proton conductivity, methanol permeability, and mechanical properties were compared. Then, MD simulations were employed to provide insights into experimentally observed phenomena and get a molecular-level understanding of the hydrophilic structure and proton characteristics in Nafion and SPEEK membranes. The local aggregation of hydrophilic clusters and the state of hydrated complexes were investigated. Eventually, the proton diffusion abilities, including vehicular and structural diffusivities, in Nafion and SPEEK membranes were compared.

## 2. Experiment and Simulation Detail

### 2.1. Materials

**Figure 1:**
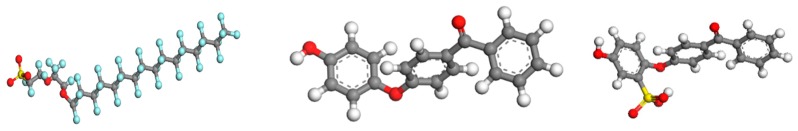
Repeat units of Nafion, poly (ether ether ketone) (PEEK) and sulfonated PEEK.

PEEK powder of VESTAKEEP4000P (density of 1.30 g cm^3^) was obtained from Degussa Co. Ltd. (Essen, Germany) Nafion solution of 20 wt% (EW = 1100) was purchased from Sigma-Aldrich Co. Ltd. (Shanghai, China) N-methyl–2-pyrrolidone (NMP) was provided by Tianjin Kemiou Chemical Reagent Co., Ltd. (Tianjin, China) Deionized (DI) water was used for all the experiments. All the reagents were of analytical grade. The repeat units of Nafion, PEEK, and sulfonated PEEK are demonstrated in Figure 1.

### 2.2. Preparation of Nafion and SPEEK Membranes

Sulfonated PEEK (SPEEK) was prepared by sulfonation of PEEK in accordance with our previous work [43]. First, 10 g PEEK was dissolved in 200 mL 98 wt % sulfuric acid (H_2_SO_4_) in a flask at room temperature. Then, the mixture was heated to 50 °C for sulfonation reaction, followed by precipitation by DI water. SPEEK with DS = 50% (equivalent weight (EW) = 657) was adopted in this research, which is proven to show the best performance among SPEEK membranes with different DS values. SPEEK (EW) = 657) and Nafion membranes (EW) = 1100) were fabricated via the solution casting method described in our previous publication [44,45]. In this study, the higher EW of Nafion than SPEEK indicated that Nafion possesses lower -SO_3_H density than that of SPEEK.

### 2.3. Characterization Methods

Water uptake (*ω*) of Nafion and SPEEK membranes was tested according to the following procedure. At 20 °C, the membranes were put into DI water for 12 h to reach the equilibrated weights (*W_wet_*). Dry mass of membranes (*W_dry_*) was measured after drying at 120 °C in vacuum oven for over 12 h. Water uptakes (ω) and the number of water molecules per -SO_3_^−^ (*λ*) were calculated according to Equations (1) and (2):

(1) $$\mathrm{Water}\;\mathrm{uptake}\;(\omega )=\frac{W_{wet}-W_{dry}}{W_{dry}}\times 100\%,$$

(2) $$\lambda (\mathrm{total})=\frac{\omega \times EW}{Mw},$$

where *W_wet_* are the weight of the wet membranes, *W_dry_* are the weight of the dry membranes, *Mw* is the molecular weight of water (18 g mol^−1^), and EW is equivalent weight of Nafion (1100 g eq^−1^) and SPEEK (657 g eq^−1^) in this study.

*λ*(total) is composed of freezable water and nonfreezable water, which were designated as *λ*(freezable) and *λ*(nonfreezable). These two were measured using differential scanning calorimetry (DSC) in a TA Q10 instrument. Membranes were pat-dried with tissue paper and immediately sealed in aluminum DSC pans. In a typical run, 4–6 mg of sample is firstly equilibrated at −60 °C and then heated to 50 °C at 5 °C min^−1^ under nitrogen atmosphere. *λ*(freezable) and *λ*(nonfreezable) were calculated according to Equations (3) and (4):

(3) $$\lambda (\mathrm{freezable})=\frac{\Delta H_{w}\times EW}{\Delta H_{f}\times M_{w}},$$

(4) λ(nonfreezable)=λ(total)−λ(freezable),

where Δ*H_w_* is obtained by integrating the area under the heating curve, Δ*H_f_* is the heat of fusion for bulk water (334 J g^−1^), and *λ*(total) is the total fraction of water in the membrane, which is the sum of freezable and nonfreezable water.

The in-plane proton conductivity was tested in a potentiostat (IviumStat). The four electrode AC impedance method was used. The conductivity was calculated as:

(5) $$\sigma =\frac{L}{R\times W\times \delta },$$

where *L* and *R* designate the distance and resistance, and *W* and *δ* denote the width and thickness of the membranes.

The methanol crossover, mechanical properties were measured in accordance with our previous publication [44].

TEM observations on the nanophase separation and hydrophilic morphology of Nafion and SPEEK membranes were obtained on a transmission electron microscope. RuO_4_ and Lead nitrate staining were used for Nafion and SPEEK membranes respectively to identify the cluster and crystallite morphology.

### 2.4. Model Constructions and Simulation Details

**Figure 2:**
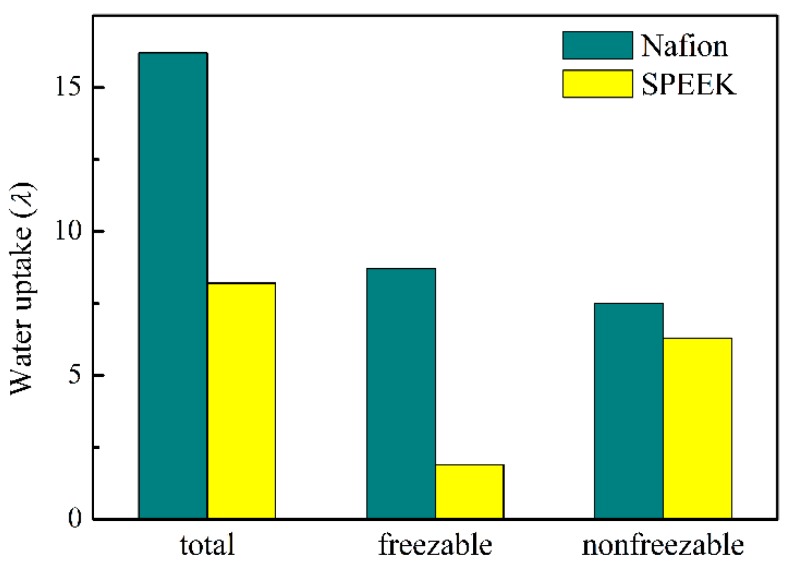
Proton conductivities of Nafion and SPEEK proton exchange membranes (PEMs) at different hydration level.

Molecular dynamics (MD) simulations were carried out to study the hydrophilic cluster morphology and the water state of Nafion and SPEEK [46]. The details were in accordance with our previous work [45]. Both Nafion chain and SPEEK chain consisted of 10 repeat units. In particular, SPEEK chain (DS = 50%) was composed of 5 sulfonated PEEK units and 5 PEEK monomers. On the basis of the water uptake results (presented in Figure 2), the *λ*(total) in Nafion and SPEEK was 16.2 and 8.2, respectively. Considering the huge difference in *λ*, comparing the properties of SPEEK and Nafion under the same *λ* is not appropriate. Therefore, in the present study, four relative water contents of 0%, 25%, 50%, and 100% for Nafion and SPEEK membranes were defined, corresponding to *λ*_Nafion_ of 0, 4, 8, and 16 and *λ*_SPEEK_ of 0, 2, 4, and 8. When the hydration level was 0, an undissociated sulfonate group (-SO_3_H) was defined, whereas a dissociated sulfonate group (-SO_3_^−^) was defined when the hydration level was above 0 [47]. The simulated densities of Nafion and SPEEK were recorded and compared with experimental values. With increasing hydration level, the reported experimental and simulated Nafion density decreased gradually from 2.05 to 1.68 g cm^−3^ [48,49,50], while the simulated density decreased from 2.07 to 1.71 g cm^−3^. The decrease in density could be attributed to the swelling of aqueous domains with increasing water content. Meanwhile, the simulated densities of SPEEK are also well coincident with the reported values [51,52,53]. These findings proved that the present simulation reproduces the density of Nafion and SPEEK perfectly.

## 3. Results and Discussion

### 3.1. Water Uptake, Proton Conductivity, Fuel Crossover and Mechanical Performance

The general performances of Nafion and SPEEK membranes are presented in Table 1. As compared to Nafion, SPEEK showed a smaller water uptake of 22.5%, and the proton conductivity in hydrated state at 80 °C is 0.120 S cm^−1^, which was substantially lower than that of the Nafion membrane. However, SPEEK exhibited extremely low methanol permeability, indicating a desired suppressed fuel crossover. In addition, a much better mechanical performance was revealed for the SPEEK membrane, which ensured good mechanical durability.

Upon water hydration, the hydrophilic cluster would become bridged proton conductive channels. Thus, the water uptake is crucial for proton conductivity. The composition of water uptake is shown in Figure 2. The SPEEK membrane possessed comparable nonfreezable water but much lower freezable water (8.7) than that of the Nafion membrane (1.9). Within sulfonated membranes, nonfreezable water was tightly bonded to the -SO_3_^−^ of the pendent side chain, whereas freezable water represented free water and loosely bonded water, which were able to diffuse combining with H_3_O^+^ through the hydrophilic domains [54,55]. Thus, the decrease in freezable water in the SPEEK membrane was unfavorable for water and proton transport. The total number of water molecules per sulfonic acid site of SPEEK was 8.2, which was considerably lower than the value of Nafion membranes (16.2). These findings suggest a relative low proton conductivity of SPEEK. Notably, the difference of water state between Nafion and SPEEK membranes might be caused by the phase separation morphology and -SO_3_H density.

**Figure 3:**
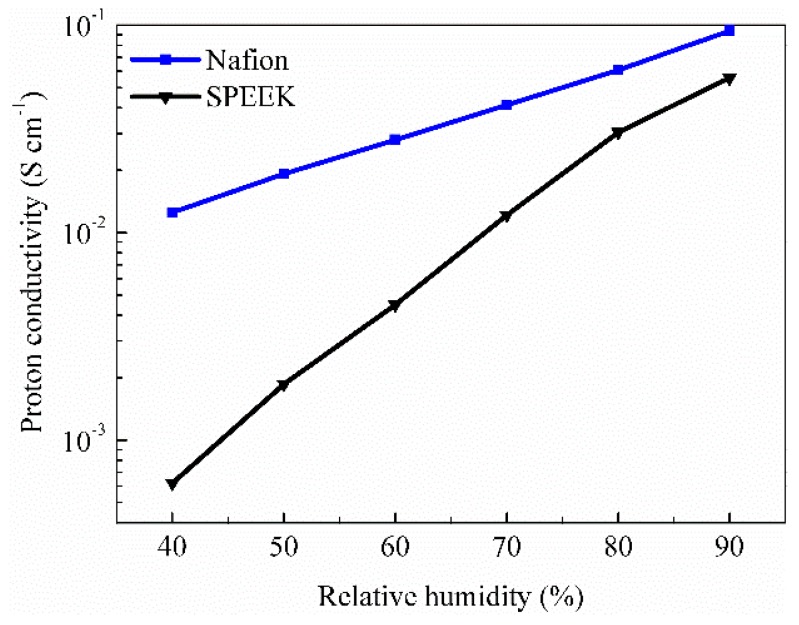
Proton conductivities of Nafion and SPEEK PEMs at different hydration levels.

The proton conductivities of the Nafion and SPEEK membranes at various relative levels of humidity (RH) ranging from 40% to 90% are shown in Figure 3. Compared with Nafion, SPEEK PEMs exhibited much lower proton conductivity, especially at low relative humidity. Employing RH = 90% and 40% as examples, the proton conductivity of Nafion was decreased from 0.094 to 0.0125 S cm^−1^, whereas the proton conductivity for SPEEK was greatly decreased from 0.056 S cm^−1^ to 6.2 × 10^−4^ S cm^−1^.

### 3.2. Nanophase Separation Morphology

**Figure 4:**
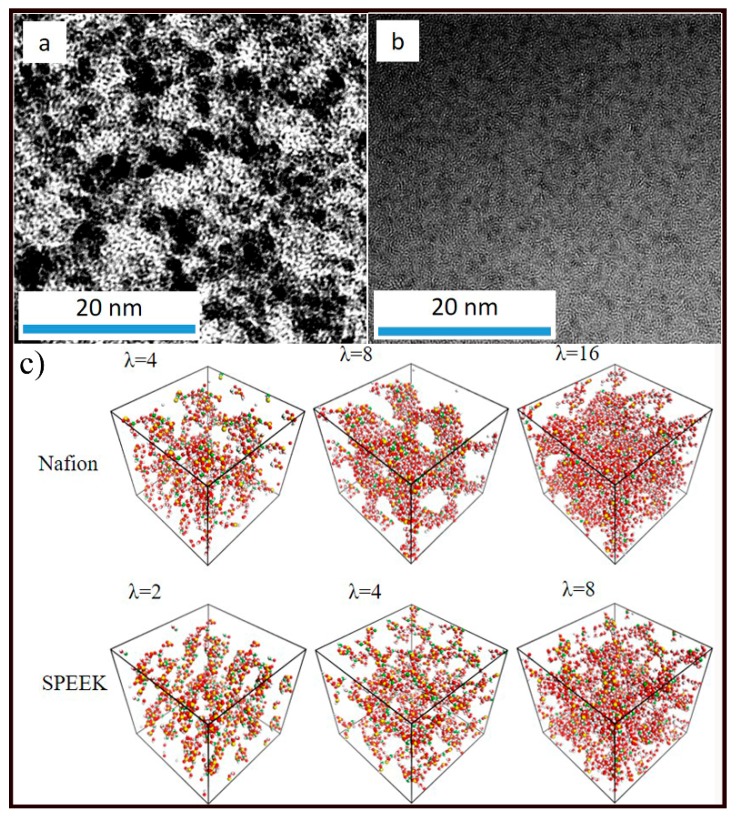
TEM images of the (**a**) Nafion membrane and (**b**) SPEEK membrane. (**c**) Final snapshots of -SO_3_^−^, H_2_O, and H_3_O^+^ in the Nafion and SPEEK matrix at 300 K. Red balls, oxygen in -SO_3_^−^ and H_2_O; green balls, oxygen in H_3_O^+^; white balls, hydrogen in H_2_O and H_3_O^+^; yellow balls, sulfur in -SO_3_^−^; other atoms were invisible.

The nanophase separation morphology of the Nafion and SPEEK membranes is shown in Figure 4a,b. Both the Nafion and SPEEK membranes represented nanophase separation morphology. Similar to the hypothesis of the Gierke model [56,57], the hydrophilic regions (dark region) were distributed in a hydrophobic matrix (white region). These hydrophilic clusters would become proton conductive channels upon water hydration, while the white matrix constituted the physical support for mechanical properties. Distinct nanophase separation and continuous clusters with a width of 3–5 nm were observed in Nafion membrane. However, the SPEEK membrane showed poor phase separation morphology, wherein separated clusters of 1 nm were found.

MD simulation of Nafion and SPEEK with different water contents was carried out. Three relative water contents of 25%, 50%, and 100% were investigated, corresponding to *λ*_Nafion_ of 4, 8, and 16 and *λ*_SPEEK_ of 2, 4, and 8. The hydrophilic groups including -SO_3_^−^, H_2_O and H_3_O^+^ are shown in Figure 4c. Meanwhile, the backbone matrix was set to be invisible to clarify the hydrophilic domain. The hydrophilic clusters were isolated at low water content. With increasing water content, neighboring hydrophilic clusters were bridged to interconnected proton conductive channels, which could facilitate both the structural and vehicular diffusion of protons. Similar to TEM images, a lower degree of nanophase separation with a smaller cluster size was observed for SPEEK. This might be attributed to the higher steric hindrance of SPEEK backbone and the lower electronegativity of H in SPEEK than that of F in Nafion.

### 3.3. Hydrophilic Cluster in Nafion and SPEEK Systems

In this section, radial distribution functions (RDFs) were carried out by MD simulations to investigate the water state in Nafion and SPEEK.

**Figure 5:**
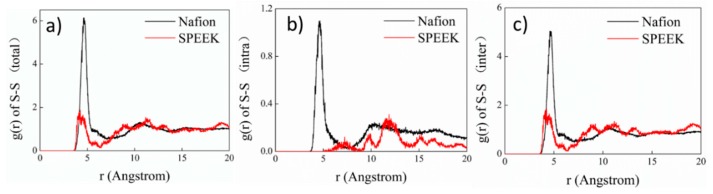
Radial distribution functions (RDFs) of the S–S pair for the Nafion and SPEEK systems (*λ* = 0). (**a**) S-S (total), (**b**) S-S (intra), (**c**) S-S (inter).

The RDF of S–S (S denotes sulfur atom in -SO_3_^−^) for Nafion and SPEEK is presented in Figure 5. As shown in Figure 5a, both RDFs of S–S (total) displayed a peak at about 4.5 Å. The peak intensity was largely ascribed to the intermolecular S–S correlation. Nafion exhibited much stronger peaks for all three types of S–S than SPEEK. In particular, the first S–S (intra) peak (4.5 Å) was missing in the RDF of SPEEK. This revealed that in SPEEK, the local aggregation of hydrophilic clusters, which was represented by sulfonate group, was poor, leading to poor cluster morphology, which agrees well with TEM results.

**Figure 6:**
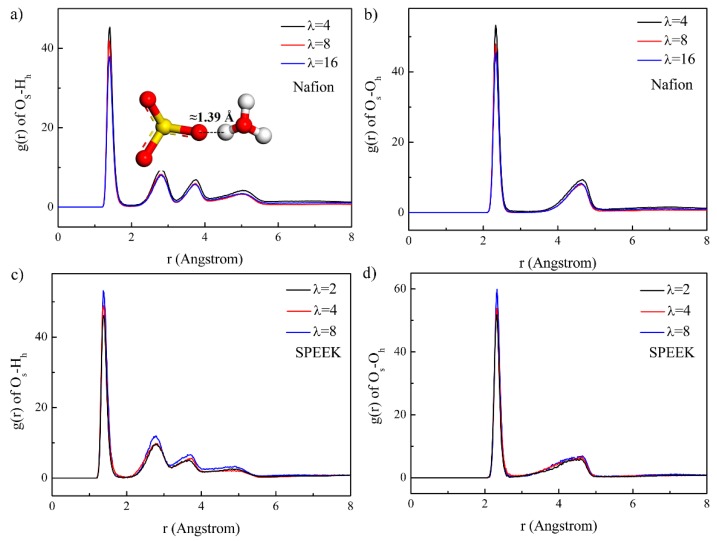
RDFs of the O_s_–H_h_ pair and O_s_–O_h_ pair for the Nafion and SPEEK systems. (**a**) O_s_-H_h_ in Nafion, (**b**) O_s_-O_h_ in Nafion, (**c**) O_s_-H_h_ in SPEEK, (**d**) O_s_-O_h_ in SPEEK.

The relations of -SO_3_^−^ and H_2_O and H_3_O^+^ were studied by the RDFs of O_s_–H_h_ and O_s_–O_h_ (O and H denote oxygen and hydrogen atom, the subscripts “s”and “h” represent -SO_3_^−^ and hydronium) in Figure 6. The RDFs of O_s_–H_h_ exhibited the first peaks at about 1.39 Å, indicating a formation of hydrogen bonding between O_s_ and H_h_ (see the inset scheme in Figure 6a). This agreed well with the reports by Piyarat et al. [58]. The RDFs of O_s_–O_h_ displayed the first peaks at 2.33 Å. As the hydration level increased, the peak intensities of O_s_–H_h_ and O_s_–O_h_ decreased for Nafion but increased for SPEEK. The corresponding average hydronium number around O_s_ was calculated and the results are listed in Table 1. It is obvious that the average hydronium number around O_s_ in SPEEK was higher than that in Nafion, indicating a stronger interaction between O_s_ and H_h_ in SPEEK, especially at high water content. This was unfavorable for proton diffusion in the hydrophilic domain.

**Figure 7:**
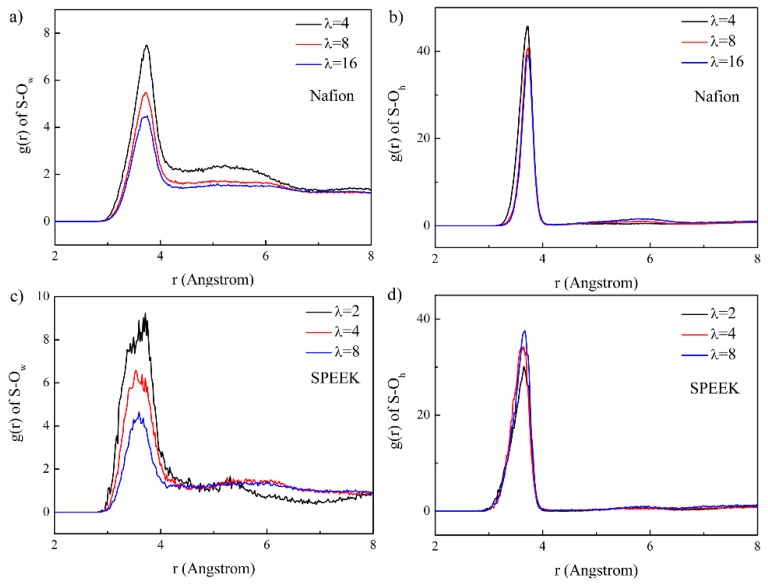
RDFs of the S–O_w_ pair and S–O_h_ pair for the Nafion and SPEEK systems. (**a**) S-O_w_ in Nafion, (**b**) S-O_h_ in Nafion, (**c**) S-O_w_ in SPEEK, (**d**) S-O_h_ in SPEEK.

The RDFs of S–O_w_ (O denotes oxygen, and “w” represents water) and S–O_h_ (O denotes oxygen, and “h” represents hydronium) for Nafion and SPEEK are presented in Figure 7. Because S atoms cannot contact H_2_O directly, the first peaks of S–O_w_ RDFs occurred at about 3.7 Å, suggesting the existence of the first hydration shell around the -SO_3_^−^. This was similar to the results reported by Zhang et al. [41]. As the hydration level increased, the peak intensity decreased. The RDFs of S–Oh displayed the first peaks at 3.7 Å, with the similar position of RDFs of S–Ow, proving that the -SO_3_^−^ attracted both water and hydronium. The hydration number of Nafion and SPEEK around the -SO_3_^−^ is displayed in Table 2. It showed that as the water content increased, the average number of H_2_O around S increased, and thus the interaction between -SO_3_^−^ and H_3_O^+^ was reduced by the water solvation of -SO_3_^−^ and more H_3_O^+^ would escape the attraction of -SO_3_^−^. Therefore, with increasing water content, the average number of H_3_O^+^ decreased. In the Nafion membrane, because of the relatively high hydration number, more H_2_O gathered around -SO_3_^−^ than in SPEEK, indicating largerproton conductive channels. It should be noted that with increasing water content, the H_3_O^+^ number in Nafion decreased considerably, whereas the H_3_O^+^ number in SPEEK decreased slightly, proving the electrostatic interaction between H_3_O^+^ and the -SO_3_^−^- in SPEEK was much stronger. As a consequence, the generation of more delocalized H_3_O^+^ in Nafion would enhance the proton conductivity.

### 3.4. Diffusion Coefficients of Water and Hydronium

**Figure 8:**
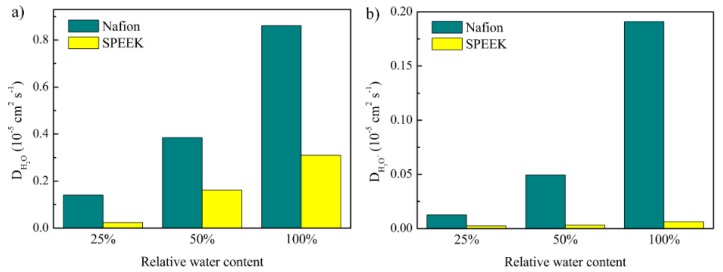
Diffusion coefficients of H_2_O and H_3_O^+^ in the Nafion and SPEEK membranes. (**a**) D_H2O_, (**b**) D_H3O+_.

On the basis of the thermodynamic movement (mean square displacement) of H_2_O and H_3_O^+^ in Nafion and SPEEK, the corresponding self-diffusion coefficients of H_2_O (D_H2O_) and H_3_O^+^ (D_H3O+_) were calculated according to Einstein relation. The simulated results at various hydration levels are shown in Figure 8.

The water cluster morphology was strongly dependent on water content. Thus, as the water content increased, the diffusion coefficients of H_2_O and H_3_O^+^ increased gradually. The simulated diffusion coefficients of H_2_O in Nafion were 0.140 × 10^−5^ cm^2^ s^−1^ for *λ* = 4 and 0.385 × 10 ^−5^ cm^2^ s^−1^ for λ = 8. These values were consistent with the experimental results of 0.184 × 10^−5^ cm^2^ s^−1^ for *λ* = 3.49 and 0.307 × 10^−5^ cm^2^ s^−1^ for *λ* = 8.77 [59]. It should be noted that because of membrane blockage, the diffusion of H_2_O in the hydrophilic domain was substantially decreased compared to that in bulk water [60]. 

Herein, only the vehicular diffusion of H_3_O^+^ was investigated, without considering Grotthuss proton hopping. When the water content increased, the diffusion coefficients of H_3_O^+^ increased, thereby promoting proton conductivity. As compared with Nafion, SPEEK exhibited extremely lower diffusion coefficients of H_2_O and H_3_O^+^, indicating a lower proton conductivity, which was consistent with the result of proton conductivity test in Figure 3. We attributed this to the worse cluster morphology (see Figure 4a) and less free H_3_O^+^ in SPEEK membranes. These findings were similar to our previous results by experimental and simulation methods [45].

The results indicated that the confinement effect of proton conductive channels on diffusion of H_2_O and H_3_O^+^ is influenced by the water content and the membranes’ morphologies.

### 3.5. Hydrated Hydronium Complexes

**Figure 9:**
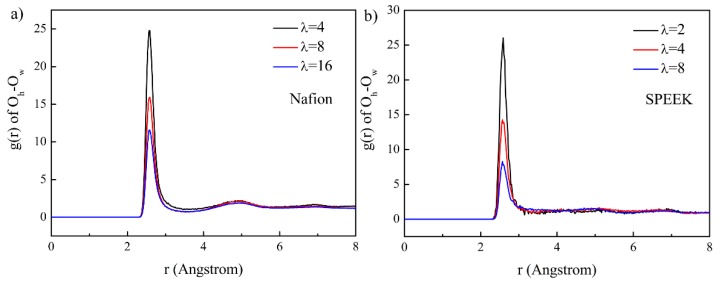
RDFs of the O_h_–O_w_ pair for the Nafion and SPEEK systems. (**a**) O_h_-O_w_ in Nafion, (**b**) O_h_-O_w_ in SPEEK.

For a particular PEM, the state of hydrated hydronium complexes was dependent on proton conductive channels, which in turn was dominated by the water content. The RDFs of O_h_ and O_w_ are shown in Figure 9. The curves can be integrated within a certain radial distance to calculate the number of H_2_O molecules (designated as n) around H_3_O^+^ to form a hydrated complexes H_3_O^+^·(H_2_O)_n_, which indicated the hydration degree of H_3_O^+^. According to Figure 9, a distance of 3.2 Å between O_h_ and O_w_ was chosen, which includes most of the H_2_O lying within the first peak around H_3_O^+^. A water number of 3.78 was reported for bulk water within 3.2 Å [61]. However, the H_2_O number around H_3_O^+^ decreased considerably because of membrane blockage. Moreover, as the water content increased, the hydration degree of H_3_O^+^ increased from 1.41 to 2.90 for Nafion and from 0.70 to 1.81 for SPEEK. A higher degree of hydration level led to abatement of electrostatic interaction between -SO_3_^−^ and H_3_O^+^, thus making it beneficial for higher vehicular transport for proton.

**Figure 10:**
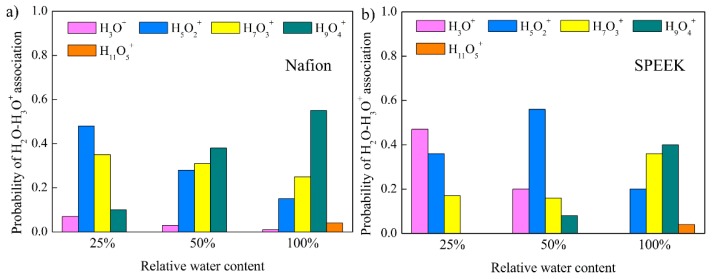
Probability of H_2_O–H_3_O^+^ association as a function of relative water content for Nafion and SPEEK. (**a**) Nafion, (**b**) SPEEK.

To further clarify the types of hydrated complexes H_3_O^+^·(H_2_O)_n_, e.g., H_3_O^+^, H_5_O_2_^+^ (denoted as Zundel ions), H_7_O_3_^+^, and H_9_O_4_^+^ (denoted as Eigen ions), the probability of H_2_O–H_3_O^+^ association as a function of relative water content for Nafion and SPEEK is shown in Figure 10. The structure of hydrated hydronium complexes would deform to adapt the confining hydrophilic channels. As the relative water content increased, the hydrophilic channels were broadened and the probability to form larger hydrated complexes increased. These results were in good accordance with published reports [41,47]. It was revealed that hydronium ions had a lower probability of being surrounded by more H_2_O molecules, proving the hydrophilic clusters were less connected in SPEEK.

It is noteworthy that the structural diffusion occurred when *n* was above 3, i.e., H_9_O_4_^+^. Particularly, with increasing relative water content, the probability of forming hydrated complexes with *n* higher than 3 increased for both Nafion and SPEEK, which contributed to higher structural diffusivity. Moreover, SPEEK exhibited much lower H_9_O_4_^+^ probability at various relative water contents, leading to lower structural diffusivity than Nafion. Combining the smaller vehicular diffusivity in SPEEK deduced from mean square displacement analysis, the total proton conduction would be much lower than that in Nafion. This was accordant with the experimental results shown in Figure 3.

## 4. Conclusions

In this work, comparative studies of the fuel cell property and hydrophilic structure of Nafion and SPEEK were performed using an experimental and simulation method. Generally, the SPEEK membrane exhibited weak phase separation and poor hydrophilic cluster morphology, leading to low water uptake and inadequate proton conductivity. Molecular dynamics (MD) simulation approaches were employed to get a molecular-level understanding of the structure–property relationship of the SPEEK and Nafion membranes. As compared with Nafion, local aggregation of hydrophilic clusters in SPEEK, represented by -SO_3_^−^, was inferior, contributing to poor proton conductive channels. Because the electrostatic interaction between O_s_ and H_h_ was much stronger in SPEEK than that in Nafion, more H_3_O^+^ were bonded to -SO_3_^−^, resulting in less delocalized free H_3_O^+^ for proton vehicular diffusion. Consequently, the diffusion coefficient of H_3_O^+^ in SPEEK (0.00628 × 10^−5^ cm^2^ s^−1^) was lower than that in Nafion (0.191 × 10^−5^ cm^2^ s^−1^). As the water content increased, the probability of forming lager hydrated complexes, i.e., H_3_O^+^·(H_2_O)_n_, increased significantly. The lower *n* value of H_3_O^+^·(H_2_O)_n_ in SPEEK suggested lower structural diffusivity than that of Nafion. Eventually, SPEEK possessed low vehicular and structural diffusivities, leading to a lower proton conductivity. This study indicated that the interaction between O_s_ and H_h_ should be carefully tuned to maintain an effective proton conduction.

## Figures and Tables

**Table 1:** The general performance of Nafion and SPEEK membranes.

PEM	Water Uptake at 20 °C	Proton Conductivity in DI Water at 80 °C (S cm^−1^)	Methanol Permeability (cm^2^ s^−1^)	Tensile Strength (MPa)	Modulus (MPa)
Nafion	26.5%	0.167	2.41 × 10^−6^	13.9	215
SPEEK	22.5%	0.120	1.97 × 10^−7^	23.9	632

**Table 2:** Hydration number around various atomic groups and diffusivity of H_2_O and H_3_O^+^ in Nafion and SPEEK.

PEM	Atomic Types	Water Content (25%)	Water Content (50%)	Water Content (100%)
Nafion	*λ*	4	8	16
	O_s_–O_h_	0.658	0.526	0.384
	S–O_w_	2.53	3.63	5.70
	S–O_h_	2.02	1.62	1.16
	O_h_–O_w_	1.41	2.26	2.90
SPEEK	*Λ*	2	4	8
	O_s_–O_h_	0.759	0.712	0.621
	S–O_w_	1.24	2.52	3.62
	S–O_h_	2.20	2.12	1.97
	O_h_–O_w_	0.70	1.14	1.81

## References

[1] Yao Z., Zhang Z., Hu M., Hou J., Wu L., Xu T. (2018). Perylene-based sulfonated aliphatic polyimides for fuel cell applications: Performance enhancement by stacking of polymer chains. J. Membr. Sci..

[2] Shahgaldi S., Alaefour I., Li X. (2018). Impact of manufacturing processes on proton exchange membrane fuel cell performance. Appl. Energy.

[3] Hibino T., Kobayashi K., Lv P., Nagao M., Teranishi S., Mori T. (2017). An Intermediate-Temperature Biomass Fuel Cell UsingWood Sawdust and Pulp Directly as Fuel. J. Electrochem. Soc..

[4] Sorte E.G., Abbott L.J., Frischknecht A.L., Wilson M.A., Alam T.M. (2018). Hydrophilic domain structure in polymer exchange membranes: Simulations of NMR spin diffusion experiments to address ability for model discrimination. J. Polym. Sci. Part B Polym. Phys..

[5] Li J., Xu G., Luo X., Xiong J., Liu Z., Cai W. (2018). Effect of nano-size of functionalized silica on overall performance of swelling-filling modified Nafion membrane for direct methanol fuel cell application. Appl. Energy.

[6] Hu H., Sui Y., Ueda M., Qian J., Wang L., Zhang X. (2018). Multi-block sulfonated poly (arylene ether nitrile) polymers bearing oligomeric benzotriazole pendants with exceptionally high H_2_/O_2_ fuel cell performance. J. Membr. Sci..

[7] Kenneth A., Robert B. (2004). State of understanding of Nafion. Chem. Rev..

[8] Li Y., Shi Y., Mehio N., Tan M., Wang Z., Hu X., Chen G.Z., Dai S., Jin X. (2016). More sustainable electricity generation in hot and dry fuel cells with a novel hybrid membrane of Nafion/nano-silica/hydroxyl ionic liquid. Appl. Energy.

[9] Hickner M.A., Ghassemi H., Kim Y.S., Einsla B.R., McGrath J.E. (2004). Alternative polymer systems for proton exchange membranes (PEMs). Chem. Rev..

[10] Wang R., Zhang W., He G., Gao P. (2014). Controlling fuel crossover and hydration in ultra-thin proton exchange membrane-based fuel cells using Pt-nanosheet catalysts. J. Mater. Chem. A.

[11] Yan X., Zhang C., Dong Z., Jiang B., Dai Y., Wu X., He G. (2018). Amphiprotic Side-Chain Functionalization Constructing Highly Proton/Vanadium-Selective Transport Channels for High-Performance Membranes in Vanadium Redox Flow Batteries. ACS Appl. Mater. Interfaces.

[12] Wu S., Qiu Z., Zhang S., Yang X., Yang F., Li Z. (2006). The direct synthesis of wholly aromatic poly (*p*-phenylene) s bearing sulfobenzoyl side groups as proton exchange membranes. Polymer.

[13] Largier T.D., Wang D., Mueller J., Cornelius C.J. (2017). Improving electrodialysis based water desalination using a sulfonated Diels-Alder poly(phenylene). J. Membr. Sci..

[14] Lee K.-S., Spendelow J.S., Choe Y.-K., Fujimoto C., Kim Y.S. (2016). An operationally flexible fuel cell based on quaternary ammonium-biphosphate ion pairs. Nat. Energy.

[15] Ono Y., Goto R., Hara M., Nagano S., Abe T., Nagao Y. (2018). High Proton Conduction of Organized Sulfonated Polyimide Thin Films with Planar and Bent Backbones. Macromolecules.

[16] Miyatake K., Chikashige Y., Higuchi E., Watanabe M. (2007). Tuned polymer electrolyte membranes based on aromatic polyethers for fuel cell applications. J. Am. Chem. Soc..

[17] Kim K., Heo P., Hwang W., Baik J.-H., Sung Y.-E., Lee J.-C. (2018). Cross-Linked Sulfonated Poly (arylene ether sulfone) Containing a Flexible and Hydrophobic Bishydroxy Perfluoropolyether Cross-Linker for High-Performance Proton Exchange Membrane. ACS Appl. Mater. Interfaces.

[18] Jiang R.C., Kunz H.R., Fenton J.M. (2005). Investigation of membrane property and fuel cell behavior with sulfonated poly(ether ether ketone) electrolyte: Temperature and relative humidity effects. J. Power Sources.

[19] Sambandam S., Ramani V. (2007). SPEEK/functionalized silica composite membranes for polymer electrolyte fuel cells. J. Power Sources.

[20] Park C.H., Lee C.H., Sohn J.-Y., Park H.B., Guiver M.D., Lee Y.M. (2010). Phase separation and water channel formation in sulfonated block copolyimide. J. Phys. Chem. B.

[21] Yan X., Sun J., Gao L., Zheng W., Dai Y., Ruan X., He G. (2018). A novel long-side-chain sulfonated poly (2,6-dimethyl-1,4-phenylene oxide) membrane for vanadium redox flow battery. Int. J. Hydrogen Energy.

[22] Elabd Y.A., Hickner M.A. (2010). Block copolymers for fuel cells. Macromolecules.

[23] O’Dea J.R., Economou N.J., Buratto S.K. (2013). Surface morphology of Nafion at hydrated and dehydrated conditions. Macromolecules.

[24] Wu L., Zhang Z., Ran J., Zhou D., Li C., Xu T. (2013). Advances in proton-exchange membranes for fuel cells: An overview on proton conductive channels (PCCs). Phys. Chem. Chem. Phys..

[25] Shin D.W., Guiver M.D., Lee Y.M. (2017). Hydrocarbon-Based Polymer Electrolyte Membranes: Importance of Morphology on Ion Transport and Membrane Stability. Chem. Rev..

[26] Peng S., Wu X., Yan X., Gao L., Zhu Y., Zhang D., Li J., Wang Q., He G. (2018). Polybenzimidazole membranes with nanophase-separated structure induced by non-ionic hydrophilic side chains for vanadium flow batteries. J. Mater. Chem. A.

[27] Park C.H., Lee S.Y., Hwang D.S., Shin D.W., Cho D.H., Lee K.H., Kim T.-W., Lee M., Doherty C.M., Thornton A.W. (2016). Nanocrack-regulated self-humidifying membranes. Nature.

[28] Kusoglu A., Weber A.Z. (2017). New Insights into Perfluorinated Sulfonic-Acid Ionomers. Chem. Rev..

[29] Thampan T., Malhotra S., Tang H., Datta R. (2000). Modeling of conductive transport in proton-exchange membranes for fuel cells. J. Electrochem. Soc..

[30] Zawodzinski T.A.J., Neeman M., Sillerud L.O., Gottesfeld S. (1991). Determination of water diffusion coefficients in perfluorosulfonate ionomeric membranes. J. Phys. Chem..

[31] Tse Y.-L.S., Herring A.M., Kim K., Voth G.A. (2013). Molecular dynamics simulations of proton transport in 3M and Nafion perfluorosulfonic acid membranes. J. Phys. Chem. C.

[32] Zhao Y.Y., Tsuchida E., Choe Y.K., Ikeshoji T., Barique M.A., Ohira A. (2014). Ab initio studies on the proton dissociation and infrared spectra of sulfonated poly (ether ether ketone) (SPEEK) membranes. Phys. Chem. Chem. Phys..

[33] Savage J., Tse Y.-L.S., Voth G.A. (2014). Proton transport mechanism of perfluorosulfonic acid membranes. J. Phys. Chem. C.

[34] Kreuer K.-D., Paddison S.J., Spohr E., Schuster M. (2004). Transport in Proton Conductors for Fuel-Cell Applications:  Simulations, Elementary Reactions, and Phenomenology. Chem. Rev..

[35] Krueger R.A., Vilčiauskas L., Melchior J.-P., Bester G., Kreuer K.-D. (2015). Mechanism of Efficient Proton Conduction in Diphosphoric Acid Elucidated via First-Principles Simulation and NMR. J. Phys. Chem. B.

[36] Telfah A., Majer G., Kreuer K.D., Schuster M., Maier J. (2010). Formation and mobility of protonic charge carriers in methyl sulfonic acid–water mixtures: A model for sulfonic acid based ionomers at low degree of hydration. Solid State Ion..

[37] Paddison S.J., Kreuer K.-D., Maier J. (2006). About the choice of the protogenic group in polymer electrolyte membranes: Ab initio modelling of sulfonic acid, phosphonic acid, and imidazole functionalized alkanes. Phys. Chem. Chem. Phys..

[38] Brandell D., Karo J., Liivat A., Thomas J. (2007). Molecular dynamics studies of the Nafion^®^, Dow^®^ and Aciplex^®^ fuel-cell polymer membrane systems. J. Mol. Model..

[39] Urata S., Irisawa J., Takada A., Shinoda W., Tsuzuki S., Mikami M. (2005). Molecular dynamics simulation of swollen membrane of perfluorinated ionomer. J. Phys. Chem. B.

[40] Elliott J.A., Paddison S.J. (2007). Modelling of morphology and proton transport in PFSA membranes. Phys. Chem. Chem. Phys..

[41] Zhang N., Liu Z., Ruan X., Yan X., Song Y., Shen Z., Wu X., He G. (2017). Molecular dynamics study of confined structure and diffusion of hydrated proton in Hyfion^®^ perfluorosulfonic acid membranes. Chem. Eng. Sci..

[42] Mabuchi T., Tokumasu T. (2018). Relationship between Proton Transport and Morphology of Perfluorosulfonic Acid Membranes: A Reactive Molecular Dynamics Approach. J. Phys. Chem. B.

[43] Du L., Yan X., He G., Wu X., Hu Z., Wang Y. (2012). SPEEK proton exchange membranes modified with silica sulfuric acid nanoparticles. Int. J. Hydrogen Energy.

[44] Wang R., Wu X., Yan X., He G., Hu Z. (2015). Proton conductivity enhancement of SPEEK membrane through n-BuOH assisted self-organization. J. Membr. Sci..

[45] Wang R., Yan X., Wu X., He G., Du L., Hu Z., Tan M. (2014). Modification of hydrophilic channels in Nafion membranes by DMBA: Mechanism and effects on proton conductivity. J. Polym. Sci. Part B Polym. Phys..

[46] Bahlakeh G., Nikazar M., Hafezi M.J., Dashtimoghadam E., Hasani-Sadrabadi M.M. (2012). Molecular dynamics simulation study of proton diffusion in polymer electrolyte membranes based on sulfonated poly (ether ether ketone). Int. J. Hydrogen Energy.

[47] Cui S., Liu J., Selvan M.E., Keffer D.J., Edwards B.J., Steele W.V. (2007). A molecular dynamics study of a Nafion polyelectrolyte membrane and the aqueous phase structure for proton transport. J. Phys. Chem. B.

[48] Weber A.Z., Newman J. (2004). Transport in Polymer-Electrolyte Membranes: II. Mathematical Model. J. Electrochem. Soc..

[49] Morris D.R., Sun X. (1993). Water-sorption and transport properties of Nafion 117 H. J. Appl. Polym. Sci..

[50] Devanathan R., Venkatnathan A., Dupuis M. (2007). Atomistic simulation of nafion membrane: I. Effect of hydration on membrane nanostructure. J. Phys. Chem. B.

[51] Komarov P.V., Veselov I.N., Chu P.P., Khalatur P.G., Khokhlov A.R. (2010). Atomistic and mesoscale simulation of polymer electrolyte membranes based on sulfonated poly (ether ether ketone). Chem. Phys. Lett..

[52] Mahajan C.V., Ganesan V. (2010). Atomistic Simulations of Structure of Solvated Sulfonated Poly (ether ether ketone) Membranes and Their Comparisons to Nafion: I. Nanophase Segregation and Hydrophilic Domains. J. Phys. Chem. B.

[53] Mahajan C.V., Ganesan V. (2013). Influence of Hydrogen Bonding Effects on Methanol and Water Diffusivities in Acid–Base Polymer Blend Membranes of Sulfonated Poly (ether ether ketone) and Base Tethered Polysulfone. J. Phys. Chem. B.

[54] Awatani T., Midorikawa H., Kojima N., Ye J., Marcott C. (2013). Morphology of water transport channels and hydrophobic clusters in Nafion from high spatial resolution AFM-IR spectroscopy and imaging. Electrochem. Commun..

[55] Schmidt-Rohr K., Chen Q. (2008). Parallel cylindrical water nanochannels in Nafion fuel-cell membranes. Nat. Mater..

[56] Gierke T.D., Munn G.E., Wilson F.C. (1981). The morphology in nafion perfluorinated membrane products, as determined by wide- and small-angle x-ray studies. J. Polym. Sci. Polym. Phys. Ed..

[57] Gierke T., Hsu W. (1982). The cluster-network nodel of ion clustering in perfluorosulfonated membranes. ACS Symp. Ser..

[58] Nimmanpipug P., Kodchakorn K., Lee V.S., Yana J., Jarumaneeroj C., Phongtamrug S., Chirachanchai S. (2018). Structural and transport phenomena of urocanate-based proton carrier in sulfonated poly (ether ether ketone) membrane composite. J. Polym. Sci. Part B Polym. Phys..

[59] Perrin J.-C., Lyonnard S., Volino F. (2007). Quasielastic Neutron Scattering Study of Water Dynamics in Hydrated Nafion Membranes. J. Phys. Chem. C.

[60] Wang J., Hou T. (2011). Application of molecular dynamics simulations in molecular property prediction II: Diffusion coefficient. J. Comput. Chem..

[61] Selvan M.E., Liu J., Keffer D.J., Cui S., Edwards B.J., Steele W.V. (2008). Molecular dynamics study of structure and transport of water and hydronium ions at the membrane/vapor interface of Nafion. J. Phys. Chem. C.

